# Impact of body fat changes in mediating the effects of antiretroviral therapy on blood pressure in HIV-infected persons in a sub-Saharan African setting

**DOI:** 10.1186/s40249-016-0152-7

**Published:** 2016-06-01

**Authors:** Chidozie U. Nduka, Olalekan A. Uthman, Peter K. Kimani, Abraham O. Malu, Saverio Stranges

**Affiliations:** Division of Health Sciences, Warwick Medical School, University of Warwick, Coventry, CV4 7AL UK; Warwick-Centre for Applied Health Research and Delivery (WCAHRD), Warwick Medical School, University of Warwick, Coventry, CV4 7AL UK; Centre for Applied Health Research and Delivery (CAHRD), Liverpool School of Tropical Medicine, Liverpool, L3 5QA UK; Department of Internal Medicine, Benue State University Teaching Hospital, Makurdi, Nigeria; Epidemiology and Public Health Research Unit, Department of Population Health, Luxembourg Institute of Health, Strassen, L-1445 Luxembourg

**Keywords:** Highly active antiretroviral therapy, Waist circumference, Body mass index, Blood pressure, HIV, Mediate

## Abstract

**Background:**

Previous studies of HIV-infected patients have shown significant associations between highly active antiretroviral therapy (HAART) and increased blood pressure; however, the mechanisms involved are less clear. Therefore, we sought to investigate the potential impact of body fat changes in mediating the effects of HAART on blood pressure changes among people living with HIV.

**Methods:**

Four hundred six consenting patients (≥18 years of age) attending a tertiary HIV clinic in semi-urban Nigeria were recruited between August and November 2014 as part of a cross-sectional study. We performed bias-corrected bootstrap tests of mediation using 95 % confidence intervals (*CI*) to determine the mediating effects of body mass index and waist circumference (mediators) on the total effects of HAART exposure (primary predictor) on blood pressure (outcome), while controlling for age, sex and other potential confounders.

**Results:**

Waist circumference remained a significant partial mediator of the total effects of HAART exposure on increasing systolic blood pressure (coefficient: 1.01, 95 % *CI*: 0.33 to 2.52, 11 % mediated) and diastolic blood pressure (coefficient: 0.68, 95 % *CI*: 0.26 to 1.89, 9 % mediated) after adjusting for age, sex, smoking status, CD4 count and duration of HIV infection. No significant mediating effect was observed with body mass index alone or in combination with waist circumference after adjusting for all potential confounders.

**Conclusion:**

Waist circumference significantly mediates the effects of HAART on blood pressure in persons living with HIV, independent of the role of traditional risk factors. The use of waist circumference as a complementary body fat measure to body mass index may improve the clinical prediction of hypertension in HIV-infected patients on antiretroviral therapy.

**Electronic supplementary material:**

The online version of this article (doi:10.1186/s40249-016-0152-7) contains supplementary material, which is available to authorized users.

## Multilingual abstracts

Please see Additional file 1 for translation of the abstracts into the six official working languages of the United Nations.

## Background

Antiretroviral drugs increase blood pressure by damaging the endothelial linings of blood vessels, which in turn interferes with the production of biological markers known to regulate blood pressure [1–3]. Nonetheless, alternative mediating mechanisms may partially account for the effects of antiretroviral therapy on blood pressure. For instance, antiretroviral drugs, such as the reverse transcriptase inhibitors and protease inhibitors, may be associated with changes in body fat measures [4, 5], which in turn may be associated with increased blood pressure [6–8]. However, the biological mechanisms of these associations remain unclear.

Studies evaluating the impact of antiretroviral therapy on blood pressure changes have usually controlled for measures of relative weight (such as body mass index) and body fat distribution (such as waist circumference) using multiple regression models: blood pressure is linearly regressed on antiretroviral status while adjusting for body mass index or waist circumference [9–11]. However, covariate adjustments using regression analyses do not account for the direction of association or the mechanisms through which blood pressure changes may occur following antiretroviral treatment. Therefore, we sought to examine whether the effects of antiretroviral therapy on blood pressure may be mediated by different measures of body fat, notably body mass index and waist circumference, in people living with HIV. Assessing these alternative causal pathways could be useful in uncovering the complex epidemiology of hypertension and other cardio-metabolic disorders among people living with HIV, especially in sub-Saharan African settings, where the burden of HIV infection is highest and the increase in antiretroviral coverage is the steepest in the world [12].

## Methods

### Study design and setting

Consenting HIV-infected patients attending the HIV clinic at the Benue State University Teaching Hospital (BSUTH) in Nigeria were recruited between August and November 2014 as part of a cross-sectional study. The HIV clinic at BSUTH is one of two tertiary HIV clinics in Benue state, with approximately 30 patients in weekly attendance. Until two years ago, Benue had consistently recorded the highest prevalence of HIV across all states in Nigeria, accounting for more than 10 % of its resident population throughout the preceding decade [13]. The current prevalence estimate of HIV in Benue now stands at 5.6 %, which remains substantially higher than the national average of 3.3 % [13].

### Participants

HIV-infected patients who were no less than 18 years old, naïve or exposed to HAART and able to communicate in English or Nigerian pidgin were included in the study. We sampled consecutive patients until we had recruited 406 participants, comprising 306 patients exposed to HAART and 100 HAART-naïve patients. HIV-infected patients who were less than 18 years old, pregnant and lactating mothers, persons in the advanced clinical stages of HIV infection (patients diagnosed with opportunistic infections or AIDS-defining illnesses), patients diagnosed with antiretroviral treatment failure and those with CD4 cell counts that had been assessed more than three months prior to the commencement of the study were considered ineligible for inclusion in the study.

### Study protocol

**Fig. 1:**
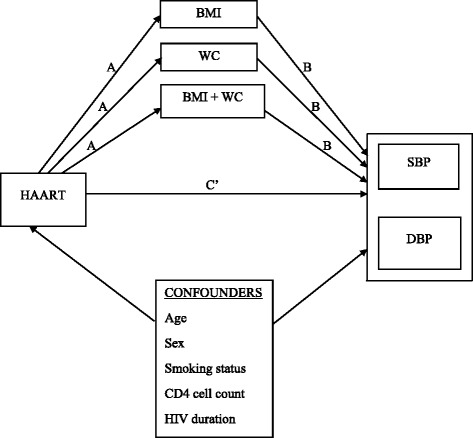
Hypothesized causal pathways explaining the effects of antiretroviral therapy (HAART) on blood pressure

Figure 1 illustrates the hypothesized mediation pathways. The primary predictor was highly active antiretroviral therapy (HAART), which was defined as treatment using two nucleoside reverse transcriptase inhibitors with either one non-nucleoside reverse transcriptase inhibitor (2NRTI + 1NNRTI) or one protease inhibitor (2NRTI + 1PI). On the other hand, patients were naïve to HAART if they had not commenced antiretroviral therapy. Body mass index and waist circumference were the mediators, and the outcomes included systolic blood pressure, diastolic blood pressure and random blood glucose levels. To adjust for potential confounding effects of the mediators, we obtained data on the traditional risk factors of high blood pressure (such as age, sex and smoking status), and HIV-related factors, including CD4 cell count and duration of HIV infection. Data on HAART status, CD4 cell counts and duration of HIV infection were obtained from the patients’ medical records. Body mass index and waist circumference were measured using standard protocols [14]. Blood pressure was measured using the Omron M10 IT Blood Pressure Monitor. The average of the first two blood pressure readings taken no less than 20 min apart was recorded as the patient’s blood pressure. A third blood pressure reading was taken in cases where the disparity was substantial (≥5 mmHg) between the first two systolic or diastolic blood pressure readings [15, 16]. All data measurements were validated and obtained similarly from all participants.

### Statistical analysis

For descriptive purposes, we summarized the distribution of the variables according to HAART status. The values were expressed as mean ± standard deviation for continuous variables and absolute numbers (percentages) for categorical variables. We stratified the association between body mass index/waist circumference and systolic/diastolic blood pressure by HAART status to rule out HAART status as an effect modifier. To investigate the potential impact of body mass index in mediating the effects of HAART on systolic and diastolic blood pressure, we estimated the mediation coefficients by fitting linear regression models following the steps outlined by Baron and Kenny: [17] in the first step, body mass index was regressed on HAART status; in the second step, systolic and diastolic blood pressure were each regressed on HAART status; in the third step, systolic and diastolic blood pressure were each regressed on body mass index while adjusting for HAART status. Using the mediation coefficients, we estimated the indirect effects (Path AB) of HAART exposure on systolic and diastolic blood pressure through body mass index. We also computed the direct effects (Path C’) of HAART exposure on systolic and diastolic blood pressure. The proportions of the total effects of HAART exposure on systolic and diastolic blood pressure that were mediated by body mass index were obtained by computing the ratios of the indirect effects to the sum of the direct and indirect effects. We subsequently controlled the mediating effects of body mass index for age, sex, smoking status, CD4 cell count and duration of HIV infection. These analyses were repeated to estimate the unadjusted and covariate-adjusted mediating effects of waist circumference and combined body mass index/waist circumference on the associations between HAART and blood pressure. Bias-corrected bootstrap tests of mediation were subsequently performed using 500 replications to determine whether the mediating effects were statistically significant using 95 % confidence intervals [18, 19]. This study is reported according to the Strengthening the Reporting of Observational Studies in Epidemiology (STROBE) statement (See Additional file 2).

### Sample size

The sample size calculation to evaluate mediation analysis for a linear model was given by the following equation: [20]

$$ n=\frac{L}{f^2}+k+1 $$

where *n* denotes the sample size; *L* is equal to 7.85, which is the linear statistic corresponding to ordinary least squares (OLS) regression for one predictor with a type I error of 0.05 and power of 0.8; *k* is equal to 1, which is the number of predictors in the OLS regression; and *f* is the coefficient of the regression equation corresponding to the indirect effect (Path AB) of the predictor variable on the outcome variable, through the mediator.

Assuming that the coefficients for Path A (0.14) and Path B (0.14) are small according to Cohen’s criteria for small effect sizes [20, 21], the indirect effect (Path AB) would entail the product of the coefficients A and B (i.e., 0.0196).

Therefore, using these default values, we aimed for a sample size of 403.

### Ethical considerations

Patients in advanced clinical stages of HIV infection, such as those with opportunistic infections or other AIDS-defining illnesses, were considered particularly vulnerable and excluded from the study. Ethical approval was obtained from the BSUTH Health Research Ethics Committee in Nigeria (NHREC/08/11/2013B), and the University of Warwick Biomedical Science Research Ethics Committee, United Kingdom (REGO-2014-711). All patients provided written informed consent prior to participation.

## Results

Table 1 summarizes the socio-demographic and clinical characteristics of the study population by HAART status. In total, 406 HIV-infected patients, comprising 306 HAART-exposed and 100 HAART-naïve subjects participated in the study. Body mass index (*P* < 0.05) and waist circumference (*P* < 0.0001) were significantly higher among HAART-exposed patients compared with HAART-naïve patients. Systolic and diastolic blood pressure levels were also significantly higher among HAART-exposed patients (*P* < 0.0001 for each). As shown in Table 2, the associations of body mass index and waist circumference with systolic and diastolic blood pressure were direct and statistically significant overall (*P* < 0.0001 for each), in HAART-exposed (*P* < 0.0001 for each), and HAART-naïve patients (*P* < 0.0001 for each).

**Table 1:** Characteristics of the study population by HAART status

Characteristics	Total (*N* = 406)	HAART-exposed (*N* = 306)	HAART-naïve (*N* = 100)
Gender			
Female, *n* (%)	278 (68.5)	200 (65.4)	78 (78.0)*
Male, *n* (%)	128 (31.5)	106 (34.6)	22 (22.0)
Age (years), mean ± SD	37.6 ± 11.2	38.6 ± 10.0	35.6 ± 12.5*
18–40 years, *n* (%)	264 (66.0)	189 (62.6)	75 (76.5)
41–64 years, *n* (%)	128 (32.0)	108 (35.8)	20 (20.4)
≥ 65 years, *n* (%)	8 (2.0)	5 (1.6)	3 (3.1)
Smoking status, *n* (%)			
Never smokers	354 (89.4)	266 (89.3)	88 (89.8)
Ever smokers	42 (10.6)	32 (10.7)	10 (10.2)
Weight (kg), mean ± SD	65.8 ± 12.8	65.6 ± 11.6	60.2 ± 12.4**
Height (cm), mean ± SD	163.1 ± 8.4	163.7 ± 8.7	160.3 ± 7.6***
BMI (kg/m^2^), mean ± SD	24.7 ± 4.6	24.5 ± 4.4	23.4 ± 4.3*
< 25 kg/m^2^, *n* (%)	245 (62.7)	175 (59.7)	70 (71.4)
≥ 25 kg/m^2^, *n* (%)	146 (37.3)	118 (40.3)	28 (28.6)
WC (cm), mean ± SD	85.9 ± 11.6	86.3 ± 10.3	82.9 ± 12.2**
CD4 count (cells/mm^3^), mean ± SD	454.1 ± 250.5	439.8 ± 255.5	504.1 ± 226.7***
< 500 cells/mm^3^, *n* (%)	252 (65.3)	200 (66.7)	52 (60.5)
≥ 500 cells/mm^3^, *n* (%)	134 (34.7)	100 (33.3)	34 (39.5)
HIV duration (months), mean ± SD	45.0 ± 29.4	50.2 ± 29.3	29.0 ± 22.8****
< 24 months, *n* (%)	138 (34.9)	88 (29.7)	50 (50.5)
24–60 months, *n* (%)	100 (25.3)	65 (22.0)	35 (35.4)
> 60 months, *n* (%)	157 (39.8)	143 (48.3)	14 (14.1)
SBP (mmHg), mean ± SD	121.2 ± 18.9	121.8 ± 19.7	112.6 ± 14.3****
DBP (mmHg), mean ± SD	77.4 ± 10.4	78.8 ± 9.9	71.6 ± 8.6****

Comparisons reserved for HAART-exposed versus HAART-naïve patients

*Abbreviations*: *BMI* body mass index, *DBP* diastolic blood pressure, *HAART* highly active antiretroviral therapy, *n* number, *SBP* systolic blood pressure, *SD* standard deviation, *WC* waist circumference

*P < 0.05

**P = 0.0001

***P < 0.01

****P < 0.00001

**Table 2:** Linear regression models of blood pressure on BMI and WC stratified by HAART status

	HAART-exposed		HAART-naive		Overall	
	Systolic BP	Diastolic BP	Systolic BP	Diastolic BP	Systolic BP	Diastolic BP
BMI	1.10 (0.60 to 1.61)	0.55 (0.29 to 0.80)	1.13 (0.50 to 1.75)	0.88 (0.52 to 1.23)	1.20 (0.79 to 1.62)	0.70 (0.48 to 0.92)
	*P* < 0.0001	*P* < 0.0001	*P* = 0.001	*P* < 0.0001	*P* < 0.0001	*P* < 0.0001
WC	0.36 (0.15 to 0.58)	0.23 (0.12 to 0.34)	0.46 (0.24 to 0.68)	0.31 (0.18 to 0.44)	0.44 (0.28 to 0.61)	0.29 (0.20 to 0.38)
	*P* < 0.0001	*P* < 0.0001	*P* < 0.0001	*P* < 0.0001	*P* < 0.0001	*P* < 0.0001

*Abbreviations*: *BMI* body mass index, *BP* blood pressure, *WC* waist circumference

### Unadjusted mediating effects of body fat measures on the association between HAART and blood pressure

Table 3 presents the unadjusted mediating effects of body mass index and waist circumference on the associations of HAART with increasing systolic and diastolic blood pressure. The indirect effects (Path AB) through body mass index were statistically significant in the associations of HAART with increasing systolic (coefficient of indirect effect: 1.29, 95 % *CI*: 0.37 to 2.70) and diastolic blood pressure (coefficient: 0.73, 95 % *CI*: 1.01 to 1.35), mediating 14 % and 10 % respectively of the total effects of HAART on systolic and diastolic blood pressure. Waist circumference mediated 15 % (coefficient: 1.35, 95 % *CI*: 0.24 to 2.76) and 12 % (coefficient: 0.87, 95 % *CI*: 0.17 to 1.69) of the total effects of HAART on systolic and diastolic blood pressure respectively. Combined body mass index/waist circumference accounted for 16 % (coefficient: 1.51, 95 % *CI*: 0.28 to 3.15) and 12 % (coefficient: 0.95, 95 % *CI*: 0.19 to 2.24) of the total effects of HAART on systolic and diastolic blood pressure levels, respectively.

**Table 3:** Unadjusted mediating effects of BMI and WC on the association between HAART and blood pressure (*N* = 406)

Mediator variable	Predictor variable	Outcome variable	Path A	Path B	Path C’	Path AB	% mediated
			Coefficient (95 % *CI*)	Coefficient (95 % *CI*)	Coefficient (95 % *CI*)	Coefficient (95 % *CI*)	
BMI	HAART	Systolic BP	1.16 (0.16 to 2.16)	1.11 (0.70 to 1.52)	8.28 (4.15 to 12.41)	1.29 (0.37 to 2.70)	14
		Diastolic BP	1.16 (0.16 to 2.16)	0.62 (0.42 to 0.83)	6.77 (4.65 to 8.88)	0.73 (1.01 to 1.35)	10
WC	HAART	Systolic BP	3.42 (0.92 to 5.92)	0.39 (0.23 to 0.56)	7.96 (3.86 to 12.06)	1.35 (0.24 to 2.76)	15
		Diastolic BP	3.42 (0.92 to 5.92)	0.26 (0.17 to 0.34)	6.71 (4.60 to 8.81)	0.87 (0.17 to 1.69)	12
Combined BMI/WC	HAART	Systolic BP	4.36 (0.85 to 7.89)	1.01 (0.24 to 1.78)	8.15 (4.08 to 12.23)	1.51 (0.28 to 3.15)	16
		Diastolic BP	4.36 (0.85 to 7.89)	0.54 (0.15 to 0.94)	6.87 (4.79 to 8.95)	0.95 (0.19 to 2.24)	12

Path A – regression coefficient for the independent variable when the mediator variable is regressed on the independent variable

Path B – regression coefficient for the mediator variable when the dependent variable is regressed on the mediator variable, adjusted for the independent variable

Path C – the direct effect of the independent variable on the dependent variable

Path AB – the indirect effect of the independent variable on the dependent variable through the mediator variable

% mediated – the amount (in percentage) of the total effect of the independent variable on the dependent variable that is attributed indirectly to the mediator variable

NS – not statistically significant at 5 % level; all other coefficients were statistically significant

95 % confidence intervals for indirect effects (Path AB) are bias-corrected

*Abbreviations*: *BMI* body mass index, *CI* confidence interval, *HAART* highly active antiretroviral therapy, *WC* waist circumference

### Covariate-adjusted mediating effects of body fat measures on the association between HAART and blood pressure

Table 4 summarizes the covariate-adjusted mediating effects of body mass index and waist circumference on the associations of HAART with increasing systolic and diastolic blood pressure. After adjusting for age, sex and smoking status, body mass index remained a significant mediator of the total effects of HAART on systolic (coefficient: 1.24, 95 % *CI*: 0.21 to 2.69, 13 % mediated) and diastolic blood pressure (coefficient: 0.68, 95 % CI: 0.30 to 1.52, 9 % mediated). Waist circumference also remained a significant partial mediator of the total effects of HAART on systolic blood pressure (coefficient: 1.07, 95 % *CI*: 0.16 to 2.74, 12 % mediated) and diastolic blood pressure (coefficient: 0.71, 95 % *CI*: 0.12 to 1.65, 10 % mediated) after adjusting for age, sex and smoking status. Similarly, the combination of body mass index with waist circumference mediated 15 % (coefficient: 1.40, 95 % *CI*: 0.33 to 3.01) and 11 % (coefficient: 0.88, 95 % *CI*: 0.26 to 1.84) of the total effects of HAART on systolic blood pressure and diastolic blood pressure respectively, after adjusting for age, sex and smoking status. However, while the mediating effects of body mass index and combined body mass index/waist circumference were attenuated by additional confounders, including CD4 cell count and duration of HIV infection, we observed that the mediating effects of waist circumference remained statistically significant, accounting for 11 % (coefficient: 1.01, 95 % *CI*: 0.33 to 2.52) and 9 % (coefficient: 0.68, 95 % *CI*: 0.26 to 1.89) of the total effects of HAART on systolic blood pressure and diastolic blood pressure, respectively.

**Table 4:** Covariate-adjusted mediating effects of BMI and WC on the association between HAART and blood pressure

Mediator variable	Predictor variable	Outcome variable	Path AB*	% mediated*	Path AB†	% mediated†	Path AB^‡^	% mediated^‡^
			Coefficient (95 % *CI*)		Coefficient (95 % *CI*)		Coefficient (95 % *CI*)	
BMI	HAART	Systolic BP	1.32 (0.30 to 2.62)	14	1.24 (0.21 to 2.69)	13	0.85 (−0.67 to 2.38)^NS^	9
		Diastolic BP	0.74 (0.20 to 1.92)	10	0.68 (0.30 to 1.52)	9	0.45 (−0.40 to 1.27)^NS^	6
WC	HAART	Systolic BP	1.14 (0.21 to 3.19)	13	1.07 (0.16 to 2.74)	12	1.01 (0.33 to 2.52)	11
		Diastolic BP	0.76 (0.16 to 1.44)	10	0.71 (0.12 to 1.65)	10	0.68 (0.26 to 1.89)	9
Combined BMI/WC	HAART	Systolic BP	1.40 (0.33 to 3.01)	15	1.40 (0.33 to 3.01)	15	1.00 (−0.40 to 3.63)^NS^	11
		Diastolic BP	0.88 (0.26 to 1.84)	11	0.88 (0.26 to 1.84)	11	0.70 (−0.18 to 2.13)^NS^	9

Path AB* – the indirect effect of HAART on the outcome variable through the mediator variable, controlled for age (continuous) and sex

Path AB† − the indirect effect of HAART on the outcome variable through the mediator variable, controlled for age (continuous), sex and smoking status

Path AB^‡^ – the indirect effect of HAART on the outcome variable through the mediator variable, controlled for age (continuous), sex, smoking status, CD4 count (continuous), and HIV duration

Coefficients for paths A, B and C’ are as shown in Table 3

% mediated* – proportion of the total effect of HAART on each outcome variable that is attributed indirectly to the mediator variable, adjusted for age and sex

% mediated† − proportion of the total effect of HAART on each outcome variable that is attributed indirectly to the mediator variable, adjusted for age, sex and smoking

% mediated^‡^ – proportion of the total effect of the independent variable on the dependent variable that is attributed indirectly to the mediator variable, adjusted for age, sex, smoking status, CD4 cell count and HIV duration

NS – not statistically significant at 5 % level; all other coefficients were statistically significant

95 % confidence intervals for indirect effects (Path AB) are bias-corrected

*Abbreviations*: *BMI* body mass index, *BP* systolic blood pressure, *CI* confidence interval, *HAART* highly active antiretroviral therapy, *WC* waist circumference

## Discussion

The main finding of this study of a sample of HIV-infected adults emphasizes the stronger impact of central fat distribution (measured by waist circumference), compared with relative weight (measured by body mass index), in mediating the associations of HAART with increased systolic and diastolic blood pressure, independent of factors that could attenuate these associations, such as age, sex, smoking status, CD4 cell count and duration of HIV infection. This finding is consistent with a large body of scientific evidence on the predominant role of central adiposity, as opposed to relative weight, in the aetiology of cardio-metabolic disorders [22–25]. We assert that the pathophysiological mechanisms to account for the differential mediating effects of body mass index and waist circumference reside mainly in the relatively limited utility of body mass index, compared with waist circumference, in detecting important cardio-metabolic changes driven by central fat accumulation [23–25]. The associations between body fat measures and blood pressure did not differ significantly between HAART-exposed and HAART-naïve patients, suggesting that HAART status may not be an effect modifier of these associations, further affirming the potential superiority of mediation modelling over effect-modification in developing alternative causal hypotheses for high blood pressure (and its complications) in persons living with HIV. Nonetheless, while the chronic inflammatory and platelet-activating effects of HIV infection ― known mechanisms that underlie blood pressure changes in antiretroviral-naïve HIV-infected patients ― corroborate this finding [26], we cannot rule out the possibility that the stratified analyses may not have been adequately powered to detect effect modification, given the relatively small sample size of HAART-naïve patients [27].

We observed that slightly higher proportions of the total effects of HAART on systolic and diastolic blood pressure were mediated by the combined effects of body mass index/waist circumference, as opposed to either body mass index or waist circumference alone, after adjusting for traditional risk factors individually (age, sex and smoking status). Previous evidence asserting that the combined measure of body mass index and waist circumference better predicts hypertension, compared with either body mass index or waist circumference, broadly corroborate this finding [28, 29].

The results of our study may have important clinical and public health implications. For instance, interventions aimed at weight management may be effective for blood pressure control among HIV-infected patients. Although lifestyle factors may be limited in predicting hypertension and other cardio-metabolic disorders in people living with HIV on HAART [30], lifestyle interventions have been shown to be effective in reducing blood pressure in this high-risk subgroup [31]. In addition, the use of waist circumference as a complementary body fat measure to body mass index may improve the clinical prediction of hypertension among HIV-infected patients on antiretroviral therapy. From a public health perspective, the prediction and early detection of high blood pressure in people living with HIV using measures of central fat distribution may potentially strengthen the surveillance of hypertension with its complications in people living with HIV, particularly in sub-Saharan African settings where cardiovascular conditions tend to be neglected in this high-risk population [32–34].

While the mediating effects of body fat measures on the association between HAART and increased blood pressure may indicate a return to health, as opposed to a pathological process, we speculate that this phenomenon would only occur in HIV-infected patients in the advanced clinical stages of HIV infection [35]. Such patients, who are likely to be antiretroviral-naïve or resistant to antiretroviral treatment, also tend to be underweight and hypotensive, such that the initiation of HAART or antiretroviral switch therapy is likely to improve body weight and blood pressure levels, albeit transiently [35–37]. However, none of the participants in our study presented with an AIDS-defining illness or were determined to be resistant to antiretroviral treatment, as specified in the exclusion criteria for participants deemed ineligible for inclusion in this study.

### Strengths and limitations

Our findings must be interpreted with caution, given the observational nature of our study which precluded any causal inferences. In addition, the potential for residual confounding and bias is high in cross-sectional studies. The present study was set in semi-urban Nigeria, which may limit the generalizability of our findings to HIV-infected populations from rural settings or outside of the sub-Saharan African region. For instance, while reverse transcriptase inhibitors are the mainstay of antiretroviral therapy in sub-Saharan African countries [32–34], protease inhibitors are the drugs of choice for initiating antiretroviral treatment among people living with HIV across European countries and the Americas [38]. Moreover, we speculate that different classes of antiretroviral drugs may confer differential effects on body fat measures and blood pressure. We did not assess the potential influence of diet and physical activity on the impact of body mass index and waist circumference in mediating the effects of HAART on blood pressure; however, lifestyle factors may be of limited value in predicting cardio-metabolic risk in people living with HIV on HAART [30]. In addition, the potential for recall bias in obtaining dietary data and self-reported physical activity is particularly high. Lastly, we could not assess the potential mediating roles of more sensitive measures of adiposity (such as fat mass) on the effects of HAART on blood pressure. The assessment of body fat mass entails the use of sophisticated and costly investigative techniques, including dual energy x-ray absorptiometry (DEXA) and bioelectrical impedance analysis [39], which are often not feasible in sub-Saharan African settings, given the existing resource constraints.

Despite these limitations, our study presents important strengths. For instance, we present the first evidence of an alternative causal pathway to explain the effects of HAART exposure on blood pressure: the independent mediating role of waist circumference on the effects of HAART on systolic and diastolic blood pressure is a novel finding of the present study. Although blood pressure was not prospectively measured upon the initiation of HAART, we affirm that the mediation models presented in this study account for the direction of association, which favours the hypothesis that HAART influences changes in body fat measures, which in turn influence changes in blood pressure. It would seem biologically less plausible if the reverse (or any other kind of interaction) was the case. Secondly, the exclusion of patients in the advanced stages of HIV-infection from the study does not allow for an alternative interpretation of the findings, for instance, the impact of body mass index and waist circumference in mediating the effects of HAART on systolic and diastolic blood pressure are more likely to be pathological than indicative of a physiological return to health. Thirdly, the inclusion of important covariates as potential confounders in the mediation analyses ensures that estimates of the mediation effects are less likely to be exaggerated. Lastly, bootstrapping with bias-corrected 95 % confidence intervals was the preferred alternative to the Sobel-Goodman mediation test in ascertaining the statistical significance of the mediating effects. Unlike the latter, bootstrapping is not affected by sample size; hence, our relatively small-sized study is unlikely to produce inaccurate estimates of indirect effects [40, 41]. In addition, the bias-corrected 95 % confidence intervals for the indirect effects corrects for potential skewedness in the data [19, 20].

## Conclusions

In conclusion, waist circumference partially mediates the effects of HAART on systolic and diastolic blood pressure, independent of covariates that could potentially attenuate these associations. Whether or not these associations are consistent in prospective analyses should be investigated by future studies.

## Abbreviations

BMI, body mass index; *CI*, confidence interval; DBP, diastolic blood pressure; DEXA, dual energy x-ray absorptiometry; HAART, highly active antiretroviral therapy; HIV, human immunodeficiency virus; SBP, systolic blood pressure; WC, waist circumference

## Additional file

Additional file 1: Multilingual abstracts in the six official working languages of the United Nations. (PDF 314 kb) Additional file 2: STROBE statement—Checklist of items that should be included in reports of cross-sectional studies. (DOCX 36 kb)
