# Supplementary material for: Impact of body fat changes in mediating the effects of antiretroviral therapy on blood pressure in HIV-infected persons in a sub-Saharan African setting
Source: Infect Dis Poverty. 2016 Jun 1;5:55. doi: 10.1186/s40249-016-0152-7 (PMC4888205; doi:10.1186/s40249-016-0152-7)
Supplement: Additional file 1: — Multilingual abstracts in the six official working languages of the United Nations. (PDF 314 kb) [file 40249_2016_152_MOESM1_ESM.pdf]

Translation of the abstract into the five official working languages of the United Nations

تأثير تغييرات دهون الجسم على تقليل تأثيرات علاج مضادات الفيروسات القهقرية على ضغط الدم في الأشخاص المصابين بفيروس نقص المناعة البشرية في موقع ما في جنوب الصحراء الأفريقية

تشيدوزي يو، ندوكا، أولاليكان أ، عثمان، بيتر ك، كيماني، أبراهام أو، مالو، سافيريو سترانجس

ملخص

خلفية: لقد أظهرت الدراسات السابقة التي أُجريت على المرضى المصابين بفيروس نقص المناعة البشرية ارتباط ملحوظ بين علاج مضادات الفيروسات القهقرية عالي النشاط (**HAART**) وبين ارتفاع ضغط الدم؛ ولكن، الآليات المتعلقة أقل وضوحاً. ولهذا، فإننا سعينا للتحقيق في التأثير المحتمل لتغييرات دهون الجسم في توسط تأثيرات مضادات الفيروسات القهقرية عالي النشاط (**HAART**) على تغييرات ضغط الدم بين الأشخاص المتعايشون مع فيروس نقص المناعة البشرية.

الطرق: أربعمائة وستة مريض متوافق (≤ 18 عاماً) يذهبون إلى عيادة من الدرجة الثالثة لمعالجة فيروس نقص المناعة البشرية في منطقة شبه حضرية في نيجيريا تم توظيفهم فيما بين أغسطس/ آب ونوفمبر/ تشرين ثاني 2014 كجزء من دراسة مستعرضة. لقد قمنا بإجراء اختبارات تمهيدية مصححة التحيز على التوسط باستخدام المهلة الزمنية الممنوحة للثقة (*CI*) والتي تبلغ 95% وذلك من أجل تحديد تأثيرات التوسط لمؤشر كتلة الجسم ومحيط الوسط (وسطاء) على التأثيرات الإجمالية للتعرض لمضادات الفيروسات القهقرية عالي النشاط (**HAART**) (المؤشرات الأولية) على ضغط الدم (النتيجة) بينما تم ضبط السن والجنس والعوامل المربكة المحتملة الأخرى.

النتائج: لقد زل محيط الوسط وسيط جزئي هام لإجمالي التأثيرات الخاصة بالتعرض لمضادات الفيروسات القهقرية على ارتفاع ضغط الدم الانقباضي (معامل: 1.01، المهلة الزمنية الممنوحة للثقة (*CI*) والتي تبلغ 95% إلى 2.52، 11% وسيطة) وضغط الدم الانبساطي (معامل: 0.68، المهلة الزمنية الممنوحة للثقة (*CI*) والتي تبلغ 95%: 0.6 إلى 1.89، 9% وسيطة) بعد تهيئة السن والجنس وحالة التدخين، عدد **CD4** ومدة الإصابة بفيروس نقص المناعة البشرية. لم يتم ملاحظة وجود تأثير هام للوساطة بمؤشر كتلة الجسم بمفرده أو مع محيط الوسط بعد تهيئة كافة العوامل المربكة المحتملة.

الاستنتاج: يعمل محيط الوسط بصورة هامة كوسيط لتأثيرات مضادات الفيروسات القهقرية على ضغط الدم في الأشخاص المتعايشون مع فيروس نقص المناعة البشرية، بناءً على دور عوامل المخاطرة التقليدية. قد يؤدي استخدام محيط الوسط كمقياس دهون الجسم مكمل لمؤشر كتلة الجسم إلى تحسين المؤشر العيادي لضغط الدم في المرضى المصابين بفيروس نقص المناعة البشرية الذين يتناولون علاج مضادات الفيروسات القهقرية.

Translated from English version into Arabic by REHAM HUSSIEN, through

TRANSLATORS
WITHOUT BORDERS

非洲撒哈拉以南地区 HIV 感染者经抗逆转录病毒疗法治疗引起的体脂变化对血压的影响

Chidozie U. Nduka, Olalekan A. Uthman, Peter K. Kimani, Abraham O. Malu, Saverio Stranges

摘要

引言：针对 HIV 感染者的研究表明，高效抗逆转录病毒疗法（HAART）与血压升高显著相关，但其机制尚不明确。因此，我们试图探讨 HIV 感染者经 HAART 治疗后体脂变化对血压变化的潜在影响。

方法：2014 年 8-11 月，在尼日利亚半城市区的一个 3 级艾滋病诊所招募了 406 名患者(≥18 岁)，患者均知情同意，进行横断面研究。在控制年龄、性别和其他潜在影响因素后，使用偏差校正的非参数百分位 Bootstrap 法(95% *CI*)确定体重指数和腰围的中位数，以确定 HAART 治疗的总疗效(主要因素)对血压的影响(结果)。

结果：在调整年龄、性别、吸烟情况、CD4 细胞数量和感染 HIV 病毒的时间等因素后，腰围仍是 HAART 治疗后的总疗效中增加收缩压（系数：1.01, 95% *CI*: 0.33-2.52, 中位数 11%）和舒张压（系数：0.68, 95% *CI*:

0.26-1.89, 中位数 9%）的一个重要的中介因素。调整了所有的潜在影响因素后，体重指数或体重指数与腰围数据相结合与血压变化未见显著的中介效应。
结论：对于经 HAART 治疗的 HIV 感染者而言，独立于传统危险因素，腰围可显著影响血压变化。经 HAART 治疗的 HIV 感染者，使用腰围作为体重指数的补充有可能提高临床上预测高血压的精确度。

Translated from English version into Chinese by Fu Yi-Jing, edited by Yang Pin, through

CHINESE CENTER FOR DISEASE CONTROL AND PREVENTION
NATIONAL INSTITUTE OF PARASITIC DISEASES

Impact des modifications de la masse graisseuse sur les effets médiés de la thérapie antirétrovirale sur la tension artérielle chez des personnes infectées par le VIH en Afrique subsaharienne

Chidozie U. Nduka, Olalekan A. Uthman, Peter K. Kimani, Abraham O. Malu, Saverio Stranges

Résumé

Contexte: Des études précédemment menées sur des patients infectés par le VIH ont montré des associations significatives entre la thérapie antirétrovirale à haute activité (HAART) et une augmentation de la tension artérielle, quoique les mécanismes impliqués ne soient pas clairement définis. Nous avons examiné l'impact potentiel des modifications de la masse graisseuse sur les effets médiés de la thérapie HAART sur la tension artérielle chez les patients infectés par le VIH.

Méthodes: Quatre cent six patients consentants (âgés de 18 ans ou plus) suivis dans une clinique de soins tertiaires contre le VIH en zone semi-urbaine au Nigeria ont été recrutés entre août et novembre 2014 dans une étude transversale. Nous avons effectué des tests bootstrap de médiation à biais corrigé en utilisant un intervalle de confiance (IC) à 95 % pour déterminer les effets médiateurs de l'indice de masse corporelle et du tour de taille (médiateurs) sur les effets totaux de l'exposition au traitement HAART (prédicteur primaire) sur la tension artérielle (résultat) en contrôlant les facteurs de confusion potentiels liés à l'âge, au sexe ou à d'autres paramètres.

Résultats: Le tour de taille est resté un médiateur partiel significatif des effets totaux de l'exposition au traitement antirétroviral HAART sur l'augmentation de la TA systolique (coefficient: 1,01; IC à 95%: 0,33 à 2,52; effets médiés à 11%) et diastolique (coefficient:0,68; IC à 95%: 0,26 à 1,89; effets médiés à 9%) après ajustements en fonction de l'âge, du sexe, de la consommation de tabac, de la numération des CD4 et de la durée de l'infection par le VIH. Aucun effet médiateur significatif n'a été observé avec l'indice de masse corporelle seul ou combiné avec le tour de taille après ajustement pour tous les facteurs de confusion potentiels.

Conclusion: Le tour de taille a un effet médiateur significatif sur les effets de la thérapie antirétrovirale HAART sur la tension artérielle chez les personnes infectées par le VIH, indépendamment du rôle des autres facteurs de risque traditionnels. Son utilisation comme méthode de mesure de masse graisseuse complémentaire de l'indice de masse corporelle peut améliorer la prédiction clinique de l'hypertension chez les patients infectés par le VIH sous thérapie antirétrovirale.

Translated from English version into French by Suzanne Assenat, through

TRANSLATORS
WITHOUT BORDERS

Влияние изменения жировых отложений в организме на опосредованное воздействие антиретровирусной терапии на кровяное давление у ВИЧ-инфицированных лиц в африканских странах к югу от Сахары

Chidozie U. Nduka, Olalekan A. Uthman, Peter K. Kimani, Abraham O. Malu, Saverio Stranges

АННОТАЦИЯ

Краткая информация: Предыдущие исследования ВИЧ-инфицированных пациентов показали значимую связь между высокоактивной антиретровирусной терапией (ВААРТ) и повышением артериального давления; однако задействованные механизмы неясны. Поэтому мы стремились исследовать потенциальное влияние изменений жировых отложений в организме на опосредованное воздействие ВААРТ на изменение кровяного давления у больных ВИЧ.

Методы: Четыреста шесть согласившихся пациентов (≥ 18 лет), посещающих районные клиники по поводу ВИЧ в частично урбанизированных районах Нигерии, были включены в исследование в период с августа по ноябрь 2014 года в рамках одномоментного исследования. Мы выполнили статические испытания опосредованного воздействия с поправкой на систематическую погрешность с доверительными интервалами 95 % (*ДИ*), чтобы определить опосредованное воздействие индекса массы тела и окружности талии (медиаторы) на общие последствия воздействия ВААРТ (первичный прогностический фактор) на артериальное давление (исход), контролируя при этом возраст, пол и другие потенциально искажающие результаты факторы.

Результаты: Окружность талии остается значительным частичным медиатором общего воздействия ВААРТ на повышение систолического артериального давления (коэффициент: 1,01, 95% *ДИ*: от 0,33 до 2,52, 11% опосредовано) и диастолического артериального давления (коэффициент: 0,68, 95% *ДИ*: 0,26 до 1,89, 9% опосредовано) после поправки на возраст, пол, статус курения, число лимфоцитов CD4 и продолжительность ВИЧ-заболевания. Никакого значительного опосредованного влияния не наблюдалось со стороны одного лишь индекса массы тела или в сочетании с окружностью талии после корректировки на все потенциально искажающие результаты факторы.

Заключение: Окружность талии значительно опосредует влияние ВААРТ на кровяное давление у больных ВИЧ, независимо от роли традиционных факторов риска. Использование окружности талии в качестве измерения жировых отложений в организме, в дополнение к индексу массы тела, может улучшить клиническое предсказание гипертензии у ВИЧ-инфицированных пациентов, проходящих курс антиретровирусной терапии.

Translated from English version into Russian by Oksana Weiss, through

TRANSLATORS
WITHOUT BORDERS

El impacto de los cambios en la grasa corporal en la mediación de los efectos que la terapia antirretroviral tiene sobre la presión sanguínea arterial en personas infectadas por VIH en un entorno de África Subsahariana

Chidozie U. Nduka, Olalekan A. Uthman, Peter K. Kimani, Abraham O. Malu, Saverio Stranges

RESUMEN

Antecedentes: Estudios anteriores de pacientes infectados por VIH han mostrado una correlación significativa entre la terapia antirretroviral altamente activa (HAART) y el incremento en la presión sanguínea arterial. Sin embargo, los mecanismos involucrados no son tan claros. Por lo tanto, quisimos investigar el impacto potencial de los cambios en la grasa corporal en la mediación de los efectos de la HAART sobre los cambios en la presión sanguínea arterial de pacientes que viven con VIH.

Métodos: Como parte de un estudio transversal, entre agosto y noviembre de 2014 se reclutaron cuatrocientos seis pacientes mayores de edad (de ≥18 años de edad) que asistieron a una clínica terciaria de VIH en una región semi-urbana de Nigeria. Llevamos a cabo pruebas de mediación bootstrap con corrección del sesgo utilizando intervalos de confianza (IC) del 95% para determinar los efectos mediadores del índice de masa corporal y perímetro de cintura (mediadores) sobre los efectos totales de la exposición a la HAART (indicador primario) en la presión sanguínea arterial (resultado), a la vez que se controlaba edad, sexo y otros causantes potenciales.

Resultados: El perímetro de cintura continuó siendo un mediador parcial de importancia sobre los efectos totales de la exposición a la HAART en el aumento de la presión sanguínea sistólica (coeficiente: 1,01, 95% *IC*: 0,33 a 2,52, 11% mediado) y la presión sanguínea diastólica (coeficiente: 0,68, 95% *IC*: 0,26 a 1,89, 9% mediado) luego de los ajustes para edad, sexo, condición de fumador, recuento de CD4 y duración de la infección por VIH. Luego de realizar los ajustes para todos los posibles causantes no se observó ningún efecto mediador significativo ya sea solo con el índice de masa corporal o en combinación con el perímetro de cintura.

Conclusión: El perímetro de cintura es un mediador significativo de los efectos de la HAART sobre la presión sanguínea arterial en personas que viven con VIH, independientemente del papel que jueguen los factores de riesgo tradicionales. El uso del perímetro de cintura como medidor adicional de la grasa corporal en el índice de masa corporal puede llegar a tener valor clínico predictivo de la hipertensión en pacientes infectados con VIH que se encuentren bajo tratamiento antirretroviral.

Translated from English version into Spanish by Maria Alejandra Aguada, through

TRANSLATORS
WITHOUT BORDERS
