# Supplementary material for: Impact of body fat changes in mediating the effects of antiretroviral therapy on blood pressure in HIV-infected persons in a sub-Saharan African setting
Source: Infect Dis Poverty. 2016 Jun 1;5:55. doi: 10.1186/s40249-016-0152-7 (PMC4888205; doi:10.1186/s40249-016-0152-7)
Supplement: Additional file 2: — STROBE statement—Checklist of items that should be included in reports of cross-sectional studies. (DOCX 36 kb) [file 40249_2016_152_MOESM2_ESM.docx]

**SUPPLEMENTAL MATERIAL**

**STROBE Statement—Checklist of items that should be included in reports of cross-sectional studies**

| **Section/Topic** | Item # | Recommendation | Reported on page # |
| --- | --- | --- | --- |
| **Title and abstract** | 1 | Body fat changes mediate the effects of antiretroviral therapy on blood pressure and blood glucose levels in people living with HIV in a sub-Saharan African setting: A mediation analysis | 1 |
|  |  | **Background:** Previous studies of HIV-infected patients show significant associations of highly active antiretroviral therapy (HAART) with worsening blood pressure and blood glucose levels; however, the mechanisms involved are less clear.  **Methods:** A sample of 406 patients (≥ 18 years) attending a tertiary HIV clinic in semi-urban Nigeria were recruited between August and November 2014 as part of a cross-sectional study. We performed bias-corrected bootstrap tests of mediation using 95% confidence intervals (CI) to determine the mediating effects of body mass index and waist circumference (mediators) on the total effects of HAART exposure (primary predictor) on blood pressure and blood glucose levels (outcomes), while controlling for age, sex and other potential confounders.  **Results:** Mediation analysis showed that waist circumference remained a significant partial mediator of the total effects of HAART exposure on increasing systolic blood pressure (coefficient: 1.01, 95% CI 0.33 to 2.52, 11% mediated), diastolic blood pressure (coefficient: 0.68, 95% CI 0.26 to 1.89, 9% mediated) and blood glucose levels (coefficient: 0.67, 95% CI 0.14 to 1.67, 25% mediated) after adjusting for age, sex, smoking status, CD4 count and duration of HIV infection. No significant mediating effect was observed with body mass index or combined body mass index/waist circumference after controlling for all potential confounders in the study.  **Conclusion:** Our findings suggest that body fat, especially central fat accumulation, significantly mediates HAART-associated increases in blood pressure and blood glucose levels among HIV-infected adults, independent of the role of traditional risk factors. | 2 |
| Introduction | | |  |
| Background/rationale | 2 | Antiretroviral drugs increase blood pressure by damaging the endothelial linings of blood vessels, which, in turn, interfere with the production of biological markers known to regulate blood pressure [1–3]. Similarly, protease inhibitors impair glucose metabolism by inhibiting the transport of glucose from the plasma into adipose tissues and muscle cells, causing raised serum glucose levels [4, 5]. Nonetheless, alternative mediating mechanisms may partly account for the effects of antiretroviral therapy on blood pressure and blood glucose levels. Of note, antiretroviral drugs, including the reverse transcriptase inhibitors and protease inhibitors, are potentially associated with body fat changes [6], which may lead to increased blood pressure and blood glucose levels [7, 8]. Studies evaluating the impact of antiretroviral therapy on blood pressure and blood glucose levels have usually been controlled for measures of relative weight, notably body mass index (BMI), and body fat distribution such as waist circumference (WC), using multiple regression analyses, such that blood pressure or blood glucose is regressed on antiretroviral status while adjusting for BMI or WC [9–11]. However, covariate adjustments using regression analyses do not account for the direction of association or the mechanisms through which blood pressure and blood glucose changes may occur following antiretroviral treatment. | 3 |
| Objectives | 3 | We aimed to examine whether the effects of highly active antiretroviral therapy (HAART) on blood pressure and blood glucose levels may be mediated by different measures of body fat, such as BMI and WC, in HIV-infected patients. We also sought to examine the combined mediating effects of BMI and WC on the impact of HAART exposure on blood pressure and blood glucose levels. | 3 |
| Methods | | |  |
| Study design | 4 | This was a cross-sectional analysis of longitudinal mediation. Ethical approval was obtained from the BSUTH Health Research Ethics Committee and the University of Warwick Biomedical Science Research Ethics Committee. | 4 |
| Setting | 5 | Consenting HIV-infected patients attending the HIV clinic at the Benue State University Teaching Hospital (BSUTH) in Nigeria were recruited between August and November 2014 as part of a cross-sectional study. The HIV clinic at BSUTH is one of two tertiary HIV clinics in Benue state, with approximately 30 patients in weekly attendance. Until two years ago, Benue had consistently recorded the highest prevalence of HIV across all states in Nigeria, accounting for more than 10% of its resident population throughout the preceding decade [12]. The current prevalence estimate of HIV in Benue now stands at 5.6%, which remains substantially higher than the national average of 3.3% [12]. | 4 |
| Participants | 6 | HIV-infected patients who were no less than 18 years old, naïve or exposed to HAART, and able to communicate in English or Nigerian pidgin were included in the study. We sampled consecutive patients until we recruited 406 participants, comprising 306 patients exposed to HAART and 100 HAART-naïve patients. HIV-infected patients who were less than 18 years old, pregnant and lactating mothers, diagnosed with AIDS-defining illnesses, antiretroviral treatment failure, or with CD4 cell counts assessed more than three months prior to commencing the study were considered ineligible for the study. | 4 |
| Variables | 7 | The primary predictor was HAART exposure, which was defined as treatment using two nucleoside reverse transcriptase inhibitors with either one non-nucleoside reverse transcriptase inhibitor (2NRTI + 1NNRTI) or one protease inhibitor (2NRTI + 1PI). On the other hand, patients were naïve to HAART if they had not commenced antiretroviral therapy. BMI and WC were the mediators, and the outcomes included systolic (SBP) and diastolic blood pressure (DBP) and random blood glucose levels. To adjust for potential confounding effects of the mediators, we obtained data on the traditional risk factors of high blood pressure and blood glucose levels (such as age, sex and smoking status), and HIV-related factors including CD4 cell count and duration of HIV infection. | 4 to 5 |
| Data sources/ measurement | 8 | The data on HAART status, CD4 cell counts and duration of HIV infection were obtained from the patients’ medical records. BMI and WC were measured using standard protocols [13]. Blood pressure was measured using the Omron M10 IT Blood Pressure Monitor. The average of the first two blood pressure readings taken no less than 20 minutes apart was recorded as the patient’s blood pressure. A third blood pressure reading was taken in cases where the disparity was substantial (≥ 5 mmHg) between the first two systolic (SBP) or diastolic blood pressure (DBP) readings [14, 15]. Blood glucose measurements were obtained using the Accu-Check Aviva Nano Blood Glucose System, United Kingdom. All data measurements were validated and obtained similarly from all participants. | 5 |
| Bias | 9 | All data measurements were validated and obtained similarly from all participants to minimize information bias. | 5 |
| Study size | 10 | The sample size calculation to evaluate mediation analysis for a linear model was given by the following equation [19]:  *n* = *L/f^2^* + *k* + 1  where *n* denotes the sample size; $L$ is equal to 7.85 which is the linear statistic corresponding to ordinary least squares (OLS) regression for one predictor with a type I error of 0.05 and power of 0.8; $k$ is equal to 1 which is the number of predictors in the OLS regression; and $f$ is the coefficient of the regression equation corresponding to the indirect effect (path AB) of the predictor variable on the outcome variable, through the mediator.  Assuming that the coefficients for paths A (0.14) and paths B (0.14) are small according to Cohen’s criteria for small effect sizes [19, 20], the indirect effect (path AB) would entail the product of the coefficients A and B (i.e. 0.0196).  Therefore, using these default values, we aimed for a sample size of 403. | 6 to 7 |
| Statistical methods | 11 | For descriptive purposes, we summarised the distribution of the variables according to HAART status. The values were expressed as mean ± standard deviation for continuous variables and absolute numbers (percentages) for categorical variables. Spearman’s correlation coefficients were computed to ascertain the relationships between the potential mediators (BMI and WC) and the study outcomes (SBP, DBP and blood glucose). To investigate the potential mediating effects of BMI on the associations of HAART exposure with increasing blood pressure and blood glucose levels, we estimated the mediation coefficients by fitting linear regression models following the steps outlined by Baron and Kenny [16]: BMI was regressed on HAART status in the first linear regression equations; the outcomes (SBP, DBP and blood glucose) were each regressed on HAART status in the second regression equations; SBP, DBP and blood glucose were each regressed on BMI, adjusting for HAART status in the third regression equations. Using the mediation coefficients, we estimated the indirect effect (Path AB) of HAART exposure on each outcome variable through BMI. We also estimated the direct effect (Path C’) of HAART exposure on each outcome variable. The proportion of the total effect of HAART exposure on each outcome that was mediated by BMI was obtained by computing the ratio of the indirect effect to the sum of the direct and indirect effects. The mediating effects of BMI were controlled for age, sex, smoking status, CD4 cell count and duration of HIV infection by adjusting the indirect effects for these covariates. These analyses were repeated to estimate the unadjusted and covariate-adjusted mediating effects of WC and combined BMI/WC. Bias-corrected bootstrap tests of mediation were subsequently performed using 500 replications to determine whether the mediation effects were statistically significant at 5% level [17, 18]. | 5 to 6 |
| Results | | |  |
| Participants | 12 | In total, 406 HIV-infected patients, comprising 306 HAART-exposed and 100 HAART-naïve subjects participated in the study. | 8 |
| Descriptive data | 13 | Table 1 summarizes the socio-demographic and clinical characteristics of the study population by HAART status. BMI (*P* < 0.05) and WC (*P* < 0.0001) were significantly higher among HAART-exposed patients compared with HAART-naïve patients. | 8 |
| Outcome data | 14 | SBP and DBP levels were also significantly higher among HAART-exposed patients (*P* < 0.0001 for each). We found no significant difference by HAART status in blood glucose levels. The correlation coefficients between the mediators and the outcomes are summarized in Table 2. Correlations of BMI (*p* < 0.0001 for each) and WC (*p* < 0.0001 for each) with SBP and DBP were positive and significant. The correlation between BMI and blood glucose level was also positive but not statistically significant, whereas, WC was directly related to blood glucose level (*p* = 0.01). | 8 |
| Main results | 15 | Table 3 presents the unadjusted mediating effects of BMI and WC on the associations of HAART exposure with increasing blood pressure and blood glucose levels. The indirect effects (Paths AB) through BMI were statistically significant for the associations between exposure to HAART and increasing SBP (coefficient of indirect effect 1.29, 95% CI 0.37 to 2.70), DBP (coefficient 0.73, 95% CI 1.01 to 1.35) and blood glucose (coefficient 1.05, 95% CI 0.02 to 3.66) levels, mediating 14%, 10% and 22% of the total effects of HAART on these outcomes respectively. WC mediated 15% (coefficient 1.35, 95% CI 0.24 to 2.76), 12% (coefficient 0.87, 95% CI 0.17 to 1.69) and 32% (coefficient 0.97, 95% CI 0.12 to 2.82) of the total effects of HAART exposure on increasing SBP, DBP and blood glucose levels respectively. Combined BMI/WC accounted for 16% (coefficient 1.51, 95% CI 0.28 to 3.15) and 12% (coefficient 0.95, 95% CI 0.19 to 2.24) of the total effects of HAART on SBP and DBP levels respectively, whereas its mediating effect on HAART-associated increase in blood glucose levels was not statistically significant (coefficient 0.90, 95% CI -0.07 to 2.31).  Table 4 summarises the covariate-adjusted mediating effects of BMI and WC on the associations between HAART exposure and increasing blood pressure and blood glucose levels. After adjusting for age, sex and smoking status, BMI remained a significant mediator of HAART-associated increases in SBP (coefficient 1.24, 95% CI 0.21 to 2.69, 13% mediated) and DBP (coefficient 0.68, 95% CI 0.30 to 1.52, 9% mediated) levels, whereas its mediating effect on HAART-associated increase in blood glucose level was attenuated (coefficient 0.33, 95% CI -0.20 to 3.10). WC remained a significant partial mediator of HAART-associated increases in SBP (coefficient 1.07, 95% CI 0.16 to 2.74, 12% mediated), DBP (coefficient 0.71, 95% CI 0.12 to 1.65, 10% mediated) and blood glucose (coefficient 0.72, 95% CI 0.06 to 2.16, 26% mediated) levels after adjusting for age, sex and smoking status. Combined BMI/WC accounted for 15% (coefficient 1.40, 95% CI 0.33 to 3.01), 11% (coefficient 0.88, 95% CI 0.26 to 1.84) and 24% (coefficient 0.65, 95% CI -0.24 to 2.15) of the total effects of HAART exposure on increasing SBP, DBP and blood glucose levels respectively, after adjusting for age, sex and smoking status.  Importantly, while the mediating effects of BMI and combined BMI/WC were attenuated by additional confounders, including CD4 cell count and duration of HIV infection, we observed that WC mediated 11% (coefficient 1.01, 95% CI 0.33 to 2.52), 9% (coefficient 0.68, 95% CI 0.26 to 1.89) and 25% (coefficient 0.67, 95% CI 0.14 to 1.67) of the total effects of HAART exposure on increasing SBP, DBP and blood glucose levels, respectively (Table 4). | 8 to 9 |
| Discussion |  |  |  |
| Key results | 16 | The main finding of this study of a sample of HIV-infected adults emphasizes the stronger effect of central fat distribution (measured by WC), as compared with BMI as a measure of relative weight, in mediating HAART-associated increases in blood pressure and blood glucose levels, independently of traditional cardiovascular risk factors and HIV-related characteristics that could attenuate these associations. This finding is consistent with a large body of scientific evidence on the predominant role of central adiposity, as opposed to relative weight, in the epidemiology of cardio-metabolic disorders [21–25].  Importantly, while the difference between the correlation coefficients of BMI and WC, in relation to blood glucose concentration, plausibly explains the differential indirect effects (unadjusted and adjusted) of BMI and WC in mediating HAART-associated increase in blood glucose level, we affirm that the pathophysiological mechanisms to account for the differential mediating effects of BMI and WC reside mainly in the relatively limited measure of BMI, compared with WC, in detecting important metabolic changes driven by central fat accumulation [23–25].  In addition, we find that slightly higher proportions of the effects of HAART exposure on systolic and diastolic blood pressure are mediated by the combined effects of BMI and WC, as opposed to either BMI or WC alone, even after adjusting for traditional risk factors. Previous evidence suggest that the combined measure of BMI and WC better predicts hypertension and diabetes mellitus, as compared with BMI or WC alone, which broadly corroborate this finding [26, 27]. | 10 to 11 |
| **Limitations** |  |  |  |
| Interpretation | 17 | Our findings must be interpreted with caution, given the observational nature of our study which precluded any causal inferences. In addition, the potential for residual confounding and bias is high in cross-sectional studies. The present study was set in semi-urban Nigeria, which might limit the generalizability of our findings to HIV-infected populations from rural settings or outside the sub-Saharan African region. We did not assess the potential influence of diet on the mediating roles of BMI and WC on HAART-associated blood pressure and blood glucose changes; however, evidence suggests that dietary factors may be of limited value in predicting HAART-associated cardio-metabolic risk [28]. Lastly, we could not assess the potential mediating roles of more sensitive measures of adiposity (such as fat mass) on the effects of HAART exposure on blood pressure and blood glucose levels. The assessment of body fat mass entails the use of sophisticated and costly investigative techniques including dual energy x-ray absorptiometry (DEXA) and bioelectrical impedance analysis [37], which are often not feasible in these settings, given resource constraints.  Despite these limitations, our study presents important strengths. For instance, we present the first evidence of alternative causal pathways to explain the effects of HAART exposure on cardio-metabolic traits: the potential mediating roles of BMI and WC on the associations of HAART with increasing blood pressure and blood glucose levels are novel findings of the present study. Secondly, the exclusion of patients in the advanced stages of HIV-infection from the study does not allow for an alternative interpretation of the findings, for instance, the mediation effects of BMI and WC on HAART-associated increases in blood pressure and blood glucose levels are more likely to be pathological than indicative of a physiological return to health. The inclusion of important covariates as potential confounders in the mediation analyses ensures that estimates of the mediation effects are less likely to be exaggerated. Bootstrapping was the preferred alternative to the Sobel-Goodman mediation test in ascertaining the statistical significance of the medication effects. Unlike the latter, bootstrapping is not affected by sample size, hence, our relatively small-sized study is unlikely to produce inaccurate estimates of the indirect effects [38, 39]. Lastly, the 95% confidence intervals of the indirect effects were bias-corrected, as opposed to percentile confidence intervals, indicating that skewness in the data was corrected [19, 20]. | 12 to 13 |
| Generalisability | 18 | The present study was set in semi-urban Nigeria, which might limit the generalizability of our findings to HIV-infected populations from rural settings or outside the sub-Saharan African region. For instance, while reverse transcriptase inhibitors are the mainstay of antiretroviral therapy in sub-Saharan African countries [30–32], protease inhibitors are the drugs of choice for initiating antiretroviral treatment among people living with HIV across European countries and the Americas [36]. Such differences in HAART regimens may modify the mediating effects of body fat measures [5–8]. | 13 |
| Other information |  |  |  |
| Funding | 19 | The University of Warwick Scholarship (Warwick ID 1160088).  FAS Marie Curie International PostDoc (2012–0064). |  |
